# Factors associated with first-attempt success of peripheral arterial catheterization in a pediatric intensive care unit: a prospective observational study

**DOI:** 10.3389/fped.2026.1802930

**Published:** 2026-04-23

**Authors:** Jiajia Li, Min Zhou, Qianqian He, Kunhui Fu, Qiaoxia Wang, Qi Dong, Ying Gu, Linjuan Wang

**Affiliations:** 1 Department of Pediatric Intensive Care, Shenzhen Children’s Hospital, Shenzhen, China; 2 Department of Nursing, Children’s Hospital of Fudan University, Shanghai, China

**Keywords:** peripheral arterial catheterization, first-attempt success, pediatric intensive care unit, risk factors, ultrasound guidance, critically ill children, vascular access

## Abstract

**Background:**

Peripheral arterial catheterization (PAC) is widely used in pediatric intensive care units (PICUs) for continuous hemodynamic monitoring and arterial blood sampling. However, achieving successful arterial access in critically ill children remains technically challenging, and evidence regarding factors associated with first-attempt success is limited.

**Methods:**

A prospective observational cohort study was conducted in a tertiary PICU. Critically ill children who underwent PAC between April 2024 and May 2025 were included. Each catheterization procedure was treated as the unit of analysis, with clustering at the patient level. Patient-, disease-, procedure-, and peri-procedural variables were collected. The primary outcome was first-attempt success, defined as successful arterial catheterization within a single skin puncture at the intended site with limited needle redirection, resulting in a functional arterial line suitable for continuous blood pressure monitoring or arterial blood gas sampling. Factors associated with first-attempt success were evaluated using multivariable generalized estimating equation (GEE) logistic regression models.

**Results:**

A total of 320 PAC procedures were analyzed, with an overall first-attempt success rate of 65% (208/320). In multivariable GEE logistic regression analysis, insertion site and catheterization technique were independently associated with first-attempt success. Compared with radial artery catheterization, cannulation at the dorsalis pedis artery (OR 0.41, 95% CI 0.20–0.83), and ulnar artery (OR 0.35, 95% CI 0.13–0.98) was associated with lower odds of success. Ultrasound-guided catheterization was associated with higher odds of first-attempt success compared with the blind technique (OR 2.10, 95% CI 1.08–4.08). Using early ultrasound rescue at the second attempt as the reference, ultrasound introduced after the fourth attempt was associated with a higher number of cannulation attempts (IRR 3.81, 95% CI 2.05–7.08).

**Conclusion:**

First-attempt success of PAC in critically ill children is influenced by both puncture site and catheterization technique. Preferential use of the radial artery and ultrasound guidance may improve cannulation success in the PICU. Early adoption of ultrasound guidance after failed landmark-guided attempts may help reduce repeated cannulation attempts.

## Introduction

Peripheral arterial catheterization (PAC) is a common and essential invasive procedure in the management of critically ill children in PICU, primarily used for continuous arterial blood pressure monitoring and repeated arterial blood gas sampling (1). However, failed catheterization or multiple puncture attempts can prolong procedure time and increase the risk of complications, including hematoma, thrombosis, infection, and arterial spasm, potentially compromising the quality of hemodynamic monitoring and adversely affecting patient outcomes (2).

Previous studies have shown that ultrasound guidance significantly improves first-attempt success rates and reduces procedure-related complications in arterial catheterization (3, 4). However, procedural success is not determined by puncture technique alone. Both patient-related factors, including age, systolic blood pressure, and body mass index, and procedure-related factors, such as puncture site selection, palpation technique, and operator experience, may also influence catheterization success (5, 6).

Despite the clinical importance of PAC in critically ill children, existing studies investigating factors associated with procedural success remain limited. Most available evidence focuses on isolated variables, such as ultrasound guidance or comparisons between puncture techniques, rather than providing a comprehensive evaluation of the combined effects of patient- and procedure-related factors (7, 8). Therefore, this prospective observational cohort study aimed to identify factors associated with first-attempt success of PAC in critically ill children in the PICU, with the goal of informing individualized catheterization strategies and improving procedural success.

## Methods

### Study design and setting

This study was a prospective observational cohort study conducted in the PICU of a tertiary children's hospital in Shenzhen, China. Data were prospectively collected between April 2024 and May 2025 as part of an ongoing evidence-based practice improvement project focusing on PAC in critically ill children. Each catheterization procedure was treated as the unit of analysis.

Clinical and procedural data were recorded contemporaneously at the time of arterial catheterization by trained members of the study team using standardized data collection forms. Data included patient demographics, clinical characteristics, catheterization site, catheter size, catheterization technique, and procedure-related variables. Only procedures with complete clinical data were included in the analysis. The study was designed and reported in accordance with the Strengthening the Reporting of Observational Studies in Epidemiology (STROBE) statement.

### Participants

Inclusion Criteria: Patients were eligible for inclusion if they met all of the following criteria: ①Admission to the PICU and receipt of PAC during hospitalization; ② Age between 1 month and 18 years at the time of catheterization; ③Complete procedural data and outcomes were recorded at the time of catheterization.

Exclusion Criteria: Patients were excluded if any of the following criteria were present: ① Arterial catheterization involving non-peripheral arteries, such as the femoral artery or other central arteries; ② Known severe coagulation disorders or arterial malformations; ③ Arterial catheterization performed solely for short-term intraoperative monitoring, procedures lacking standardized data collection, or catheters inserted at outside institutions prior to admission; ④ Procedures performed under emergency resuscitation conditions in which procedural outcomes could not be reliably assessed.

### Catheterization procedure

PICU nurses who had received institutional accreditation for arterial puncture performed PAC. Completing rigorous theoretical and procedural tests and having at least four years of PICU experience were prerequisites for certification. About 30 to 50 arterial procedures were completed each month by certified nurses who regularly performed arterial blood gas sampling and arterial catheterization. Two specialized nurses with training and certification in ultrasound-guided vascular access carried out the ultrasound-guided catheterization. 10–15 arterial catheterizations were executed by these operators per month, in addition to approximately 20–30 ultrasound-guided vascular access procedures. Catheterization was conducted using a short-axis approach, and ultrasound examinations were conducted using a portable ultrasound system (LOGIQ e, GE Healthcare, Chicago, IL, USA) outfitted with a high-frequency linear probe during the study period.

Before catheterization, the patient's vascular conditions, contraindications, and indications were evaluated. The catheter size was determined primarily by the patient's body weight for landmark-based catheterization (9), while it was selected based on vascular conditions for ultrasound-guided catheterization, typically corresponding to approximately two-thirds of the target arterial diameter. The patients were positioned supine with the forearm supinated and the wrist extended by approximately 30 to 45 degrees. The technique for arterial puncture was either palpation-guided or ultrasound-guided. While the ultrasound approach showed the vessel and needle tip in real time under a short-axis view, the palpation method detected the artery by feeling the arterial pulsation. The angle at which the needle was inserted was roughly 30 to 45 degrees. The insertion angle was reduced and the needle was advanced an additional 1 to 2 mm to ensure that the catheter was entirely within the vessel after pulsatile blood return confirmed entry into the arterial lumen. Subsequently, the catheter was advanced to completing the cannulation. A disposable intravenous catheter (Introcan Safety® Winged-PUR, B. Braun, Melsungen, Germany) was used to insert arterial catheters. Correct catheter placement and successful cannulation were confirmed by the appearance of a typical arterial pressure waveform, and the catheter was fixed with a clear dressing.

### Variables and data collection

Based on the study objectives and prior literature, variables potentially associated with PAC outcomes were systematically collected and categorized into patient-related characteristics, disease-related factors, procedure-related factors, and peri-procedural clinical status.

### Patient-Related characteristics

Patient-related variables included sex, age, and body mass index (BMI). BMI was calculated using the most recent height and weight measurements obtained within 3 days prior to arterial catheterization.

### Disease-Related factors

Disease-related variables included primary diagnosis, illness severity score, and coagulation parameters. Primary diagnoses were classified according to the major acute clinical condition at PICU admission. Classification was independently performed by two qualified physicians based on medical record review, and discrepancies were resolved by consensus. Illness severity was quantified using the Pediatric Index of Critical Illness Score (PICS) (10). Physiologic parameters required for score calculation were obtained from records documented within 24 h after hospital or PICU admission. Coagulation parameters included platelet count (PLT), international normalized ratio (INR), and activated partial thromboplastin time (APTT). The most recent laboratory results obtained prior to the arterial catheterization procedure were recorded. If no relevant laboratory data were available within 24 h before the procedure, the value was treated as missing.

### Procedure-Related factors

Procedure-related variables included puncture site, catheter size, and puncture technique. Puncture technique was categorized as either ultrasound-guided catheterization, defined as real-time visualization of the target artery and needle trajectory using ultrasound equipment, or landmark-based catheterization, defined as arterial puncture performed using anatomical landmarks and palpation.

### Peri-Procedural clinical Status

Peri-procedural clinical variables included use of invasive mechanical ventilation, procedural cooperation, volume expansion, use of vasoactive agents, and blood pressure status. Procedural cooperation was defined as the patient's ability to remain sufficiently calm and cooperative during the catheterization procedure under sedation or comfort measures, allowing the puncture site to remain relatively stable. Procedural cooperation was assessed and recorded immediately after the procedure by the operator and assisting nurse. Volume expansion was defined as the rapid administration of crystalloid or colloid fluids within 1 h prior to catheterization for the purpose of improving hemodynamic status. Use of vasoactive agents was defined as the continuous intravenous infusion of any vasoactive medication at the time of catheterization to maintain circulatory stability. Blood pressure status was classified based on the mean arterial pressure measured under resting conditions prior to catheterization and interpreted using age-specific reference values.

### Outcome definition

First-attempt success of PAC was defined as successful catheter placement at the intended puncture site during a single skin puncture, resulting in a functional arterial line suitable for continuous arterial blood pressure monitoring and/or arterial blood gas sampling. Successful catheterization required fulfillment of all of the following criteria: ① Procedural efficiency: Successful catheter insertion achieved during a single skin puncture, allowing a limited number of needle redirections (up to three) without withdrawal of the needle from the skin. One attempt was defined as a single skin penetration performed to access the target artery. Needle redirection within the same puncture site without needle withdrawal was not considered an additional attempt (11). ② Technical success: Observation of free-flowing, pulsatile arterial blood return following catheter insertion. ③ Functional confirmation: Acquisition of a continuous, clearly interpretable arterial pressure waveform on the bedside monitor after correct connection to a pressurized flush system and pressure transducer. ④ Safety: Absence of major procedure-related complications requiring clinical intervention during the procedure or within 30 min after catheter placement, including but not limited to progressive hematoma (>2 cm in diameter), suspected arterial dissection, or signs of distal limb ischemia. Failure was defined as the inability to meet any of the above criteria or the requirement for additional skin punctures beyond the initial puncture.

### Statistical analysis

R software (R Foundation for Statistical Computing, Vienna, Austria) was employed to conduct all statistical analyses. During the study period, the catheter insertion event was employed as the unit of analysis due to the possibility that individual patients would endure multiple peripheral arterial catheterization procedures. Generalized estimating equations (GEE) with patient ID as the clustering variable and exchangeable correlation structures were used to account for procedure clustering among patients. Data was reported as mean ± SD or median with IQR for continuous variables, and frequencies and percentages for categorical variables. To compare groups, Wilcoxon rank-sum, Pearson *χ*², or Fisher exact tests were used as needed.

Univariable GEE logistic regression models were built to detect first-attempt success factors. Variables with *P* < .10 and clinically relevant variables were included in the multivariable model. Multicollinearity was assessed using VIF/GVIF. Poisson regression models were used to examine the relationship between ultrasonography guidance timing and catheterization attempts. All statistical tests were two-sided, and *P* < .05 was considered significant.

## Results

### Study population and catheterization characteristics

Screening was conducted on a total of 226 critically ill children who underwent 535 peripheral arterial catheterization procedures during the study period. 320 catheterization procedures were included in the final analysis after procedures with incomplete data were excluded. The median PICS score was 8.60 (IQR 8.00–9.00), and the MAP was 68.7 mmHg (IQR 59.7–79.7). Neurologic diseases (23.8%), respiratory diseases (17.5%), and hematologic diseases (12.5%) comprised the most prevalent diagnostic categories. The dorsalis pedis artery (14.7%) was the second most frequently used insertion site, following the radial artery (76.6%). Ultrasound-guided catheterization was implemented in 22.5% of the procedures. The baseline is detailed in Table 1.

**Table 1:** Baseline characteristics according to first-attempt success.

Variable	Overall (*n* = 320)	Failure (*n* = 112)	Success (*n* = 208)	*P* value
**A Continuous variables**				
Weight(kg)	19.90 (12.00, 32.00)	18.00 (10.60, 32.00)	21.85 (12.45, 32.23)	.20
PICS score	8.60 (8.00, 9.00)	8.60 (8.00, 8.85)	8.60 (8.00, 9.00)	.96
PLT(×10⁹/L)	213 (108, 306)	210 (98, 296)	213 (130, 326)	.21
INR	1.19 (1.10, 1.38)	1.21 (1.12, 1.41)	1.18 (1.10, 1.37)	.14
APTT (s)	39.6 (34.4, 48.5)	39.5 (35.0, 49.7)	39.8 (33.6, 46.7)	.48
PT (s)	15.0 (13.9–16.7)	15.3 (14.2–17.2)	14.8 (13.7–16.4)	.03
TT (s)	17.1 (15.6–19.0)	16.9 (15.5–18.8)	17.3 (15.6–19.0)	.28
Fibrinogen (g/L)	2.77 (2.04, 4.07)	2.88 (1.91, 4.07)	2.72 (2.12, 4.07)	.91
FDP(mg/L)	4.8 (2.8–13.1)	4.6 (2.8–13.2)	5.0 (2.8–12.8)	.85
D-dimer (mg/L)	1.37 (0.71, 3.37)	1.38 (0.76, 2.90)	1.37 (0.70, 3.50)	.73
MAP (mmHg)	68.7 (59.7, 79.7)	68.7 (57.3, 79.8)	68.8 (60.9, 79.4)	.46

B Categorical variables					
Variable	Level	Overall *n* (%)	Failure *n* (%)	Success *n* (%)	*P* value
Sex	Male	189 (59.1%)	63 (56.2%)	126 (60.6%)	.45
	Female	131 (40.9%)	49 (43.8%)	82 (39.4%)	
Age group	Infant	46 (14.4%)	21 (18.8%)	25 (12%)	.23
	Toddler	41 (12.8%)	17 (15.2%)	24 (11.5%)	
	Preschool	46 (14.4%)	14 (12.5%)	32 (15.4%)	
	School_age	121 (37.8%)	35 (31.2%)	86 (41.3%)	
	Adolescent	66 (20.6%)	25 (22.3%)	41 (19.7%)	
Diagnosis	Respiratory diseases	56 (17.5%)	15 (13.4%)	41 (19.7%)	.11
	Neurologic diseases	76 (23.8%)	37 (33%)	39 (18.8%)	
	Hematologic diseases	40 (12.5%)	13 (11.6%)	27 (13%)	
	Circulatory diseases	52 (16.2%)	17 (15.2%)	35 (16.8%)	
	Trauma/poisoning	25 (7.8%)	9 (8%)	16 (7.7%)	
	others	71 (22.2%)	21 (18.8%)	50 (24%)	
Insertion site	Radial artery	245 (76.6%)	71 (63.4%)	174 (83.7%)	<.001
	Dorsalis pedis artery	47 (14.7%)	23 (20.5%)	24 (11.5%)	
	Ulnar artery	19 (5.9%)	10 (8.9%)	9 (4.3%)	
	others	9 (2.8%)	8 (7.1%)	1 (0.5%)	
Catheter size	24G	152 (47.5%)	58 (51.8%)	94 (45.2%)	.26
	22G	168 (52.5%)	54 (48.2%)	114 (54.8%)	
Initial technique	Blind	248 (77.5%)	92 (82.1%)	156 (75%)	.14
	Ultrasound-guided	72 (22.5%)	20 (17.9%)	52 (25%)	
Fluid resuscitation	No	266 (83.1%)	94 (83.9%)	172 (82.7%)	.78
	Yes	54 (16.9%)	18 (16.1%)	36 (17.3%)	
Vasoactive drugs	No	198 (61.9%)	68 (60.7%)	130 (62.5%)	.75
	Yes	122 (38.1%)	44 (39.3%)	78 (37.5%)	
Mechanical ventilation	No	132 (41.2%)	38 (33.9%)	94 (45.2%)	.051
	Yes	188 (58.8%)	74 (66.1%)	114 (54.8%)	
Compliance	good	234 (73.1%)	79 (70.5%)	155 (74.5%)	.44
	poor	86 (26.9%)	33 (29.5%)	53 (25.5%)	

Continuous variables are presented as median (interquartile range).

Categorical variables are presented as *n* (%).

Group comparisons were performed using the Mann–Whitney U test for continuous variables and the *χ*² test or Fisher's exact test for categorical variables.

### Success rate

The overall first-attempt success rate was 65% (208/320). The success rate of the initial attempt varied depending on the location of insertion. The success rate for radial artery catheterization was the highest at 83.7%, while the dorsalis pedis artery and ulnar artery had lesser success rates at 51.1% and 47.4%, respectively (*P* < .001). There were no statistically significant differences in first-attempt success among age categories, sexes, catheter sizes, or peri-procedural hemodynamic variables.

### Univariable analysis of factors associated with first-attempt success

The univariable GEE logistic regression analysis results are represented in Table 2. Age group and insertion site were substantially correlated with first-attempt success among the variables that were examined. School-age children had a higher likelihood of success on their first endeavor compared to infants (OR 2.26, 95% CI 1.08–4.70, *P* = .03). In comparison to the radial artery, the dorsalis pedis artery (OR 0.42, 95% CI 0.21–0.85, *P* = .02) and other arterial sites (OR 0.07, 95% CI 0.01–0.45, *P* = .005) were associated with reduced odds of first-attempt success. The likelihood of first-attempt success was lower among patients with neurologic diseases than those with respiratory diseases (OR 0.42, 95% CI 0.19–0.91, *P* = .03), while no significant differences were observed among other diagnostic categories. A lack of significant associations was observed with respect to sex, weight, PLT, coagulation parameters, MAP, catheter size, catheterization technique, fluid resuscitation, vasoactive drug use, mechanical ventilation, and patient compliance.

**Table 2:** Univariable GEE logistic regression for factors associated with first-attempt success.

Variable	Level	OR (95%CI)	*P* value
Sex	Male (reference)	——	——
	Female	0.84 (0.51, 1.38)	.50
Age group	Infant (reference)	——	——
	Toddler	1.27 (0.51, 3.12)	.61
	Preschool	1.87 (0.74, 4.72)	.19
	School_age	2.26 (1.08, 4.70)	.03
	Adolescent	1.29 (0.60, 2.79)	.52
Weight (kg)	Per kg increase	1.01 (0.99, 1.03)	.35
Diagnosis	Respiratory diseases (reference)	——	——
	Neurologic diseases	0.42 (0.19, 0.91)	.03
	Hematologic diseases	0.79 (0.31, 2.00)	.61
	Circulatory diseases	0.76 (0.33, 1.76)	.52
	Trauma/poisoning	0.63 (0.21, 1.90)	.41
	Others diagnoses	0.85 (0.38, 1.89)	.69
PICS score	Per 10-point increase	1.04 (0.72, 1.50)	.83
PLT (×10⁹/L)	Per 50 × 10⁹/L increase	1.03 (0.95, 1.12)	.45
INR	Per unit increase	.82 (0.58, 1.16)	.26
APTT (s)	Per 5 s increase	1.01 (0.97, 1.06)	.58
PT (s)	Per 5 s increase	0.85 (0.69, 1.05)	.13
TT (s)	Per 5 s increase	0.99 (0.96, 1.03)	.77
Fibrinogen (g/L)	Per unit increase	1.03 (0.89, 1.19)	.68
FDP(mg/L)	Per 5 mg/L increase	1.00 (0.99, 1.02)	.65
D-dimer (mg/L)	Per mg/L increase	1.00 (0.99, 1.02)	.57
MAP (mmHg)	Per 10 mmHg increase	1.04 (0.91, 1.19)	.57
Insertion site	Radial artery (reference)	——	——
	Dorsalis pedis artery	0.423 (0.21, 0.85)	.02
	Ulnar artery	0.45 (0.18, 1.13)	.09
	Other sites	0.07 (0.01, 0.45)	.005
Catheter size	24G (reference)	——	——
	22G	1.30 (0.80, 2.12)	.28
Initial technique	Blind (reference)	——	——
	Ultrasound-guided	1.53 (0.88, 2.67)	.15
Fluid resuscitation	No (reference)	——	——
	Yes	1.33 (0.72, 2.45)	.38
Vasoactive drugs	No (reference)	——	——
	Yes	1.07 (0.65, 1.77)	.79
Mechanical ventilation	No (reference)	——	——
mechanical_ventilation	mechanical_ventilationYes	0.68 (0.40, 1.19)	.14
Compliance	Good (reference)	——	——
	Poor	0.78 (0.45, 1.36)	.38

### Multivariable GEE logistic regression analysis of factors associated with first-attempt success

**Figure 1:**
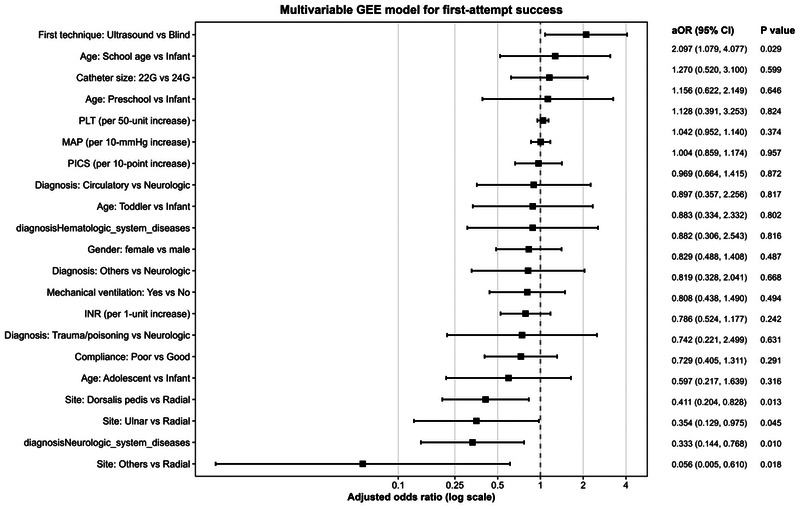
Factors associated with first-attempt success.

Table 3 illustrates the outcomes of the multivariable GEE logistic regression analysis. After adjusting for relevant confounders, insertion site, neurologic disorders, and catheterization technique independently predicted first-attempt success. The odds of first-attempt success were lower for the dorsalis pedis artery (OR 0.41, 95% CI 0.20–0.83, *P* = .01), ulnar artery (OR 0.35, 95% CI 0.13–0.98, *P* = .045), and other arterial sites (OR 0.06, 95% CI 0.01–0.61, *P* = .02) compared to radial artery catheterization. The odds of first-attempt success were lower in neurologic diseases than in respiratory diseases (OR 0.33, 95% CI 0.14–0.77, *P* = .010). Furthermore, the rate of first-attempt success was independently higher with ultrasound-guided catheterization than with the blind technique (OR 2.10, 95% CI 1.08–4.08, *P* = .03). No significant association was observed between the outcome and other variables, such as age, sex, illness severity (PICS), MAP, catheter size, platelet count, coagulation parameters, mechanical ventilation, and patient compliance. Clinically relevant factors and variables with *P* < .10 in univariable analyses were incorporated into the multivariable model. No significant collinearity was observed among the included variables in the multicollinearity diagnostics (all GVIF-adjusted values were less than 2) which is illustrated in Supplementary Table S1. Figure 1 shows a forest plot of multivariable model variables’ adjusted odds ratios and 95% confidence intervals.

**Table 3:** Multivariable GEE logistic regression for factors associated with first-attempt success.

Variable	Level	OR (95%CI)	*P* value
Insertion site	Radial artery (reference)	——	——
	Dorsalis pedis artery	0.41 (0.20, 0.83)	.01
	Ulnar artery	0.35 (0.13, 0.98)	.045
	Other sites	0.06 (0.01, 0.61)	.02
Diagnosis	Respiratory diseases (reference)	——	——
	Neurologic diseases	0.33 (0.14, 0.77)	.010
	Hematologic diseases	0.88 (0.31, 2.54)	.82
	Circulatory diseases	0.90 (0.36, 2.26)	.82
	Trauma/poisoning	0.74 (0.21, 2.50)	.63
	Other diagnosis	0.82 (0.33, 2.04)	.67
Age group	Infant (reference)	——	——
	Toddler	0.88 (0.33, 2.33)	.80
	Preschool	1.13 (0.39, 3.25)	.82
	School_age	1.27 (0.52, 3.10)	.60
	Adolescent	0.60 (0.22, 1.64)	.32
Sex	Male (reference)	——	——
	Female	0.83 (0.49, 1.41)	.49
PICS score	Per 10-point increase	0.97 (0.66, 1.42)	.87
MAP (mmHg)	Per 10-mmHg increase	1.00 (0.86, 1.17)	.96
Catheter size	24G (reference)	——	——
	22G	1.16 (0.62, 2.15)	.65
Initial technique	Blind (reference)	——	——
	Ultrasound-guided	2.10 (1.08, 4.08)	.03
Mechanical ventilation	No (reference)	——	——
	Yes	0.81 (0.44, 1.49)	.49
Compliance	Good (reference)	——	——
	poor	0.73 (0.41, 1.31)	.29
PLT (×10⁹/L)	Per 50 × 10⁹/L increase	1.04 (0.95, 1.14)	.37
INR	Per unit increase	0.79 (0.52, 1.18)	.24

### Cumulative success according to number of attempts

**Figure 2:**
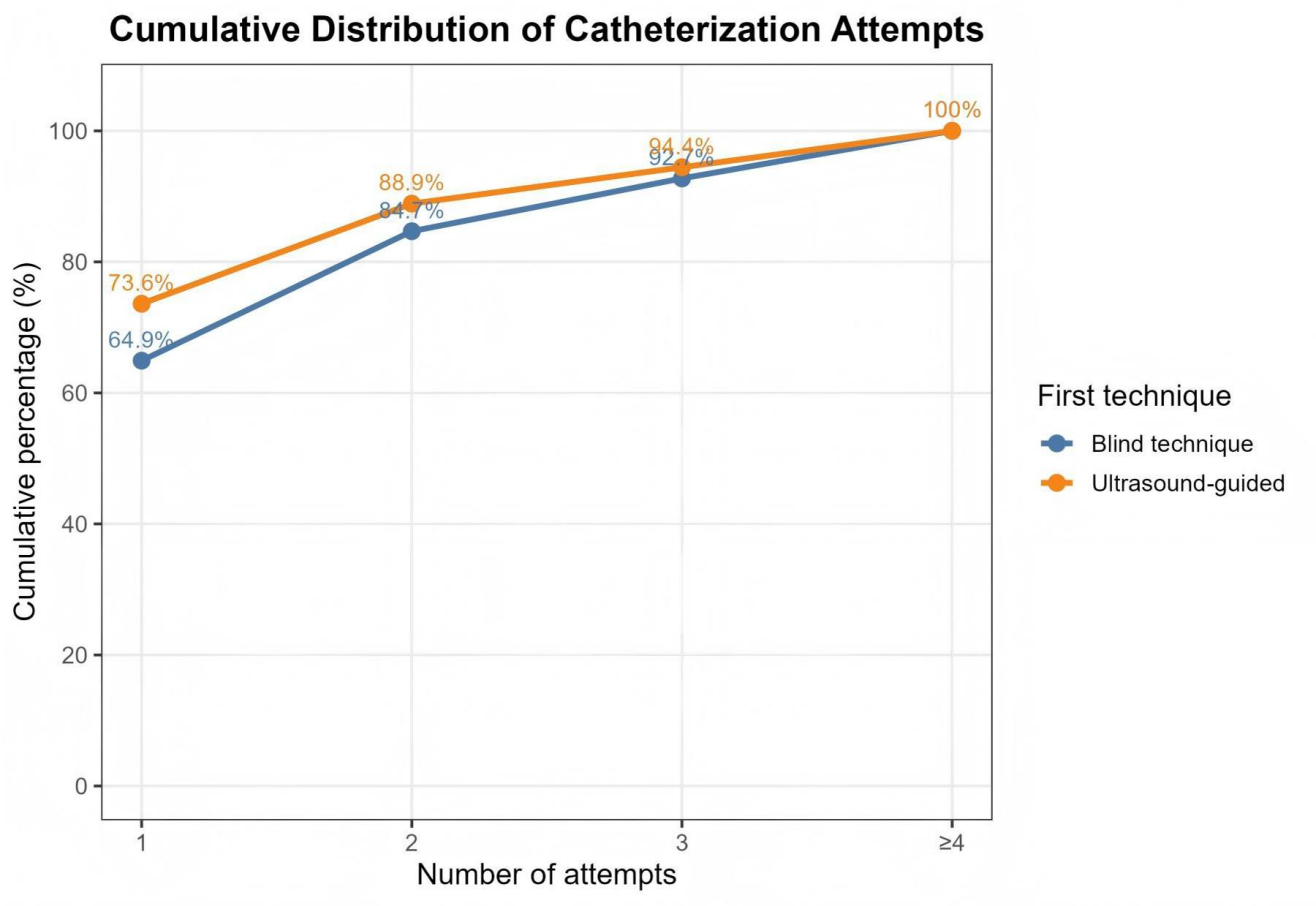
Cumulative success by number of attempts.

The cumulative success rate of both techniques, as illustrated in Figure 2, increased as the number of catheterization attempts increased. The cumulative success rates for the blind technique were 64.9% after the first attempt and 84.7% after two attempts. Compared to the first attempt, the ultrasound-guided technique obtained a higher cumulative success rate of 73.6%, and 88.9% after two attempts. Within four attempts, all procedures were successful, and the cumulative success rate in both groups exceeded 90% after three attempts. Ultrasound guidance consistently demonstrated a higher cumulative success rate across all endeavors. The distribution of catheterization attempts is illustrated in Supplementary Table S1.

### Timing of ultrasound rescue after blind failure

**Figure 3:**
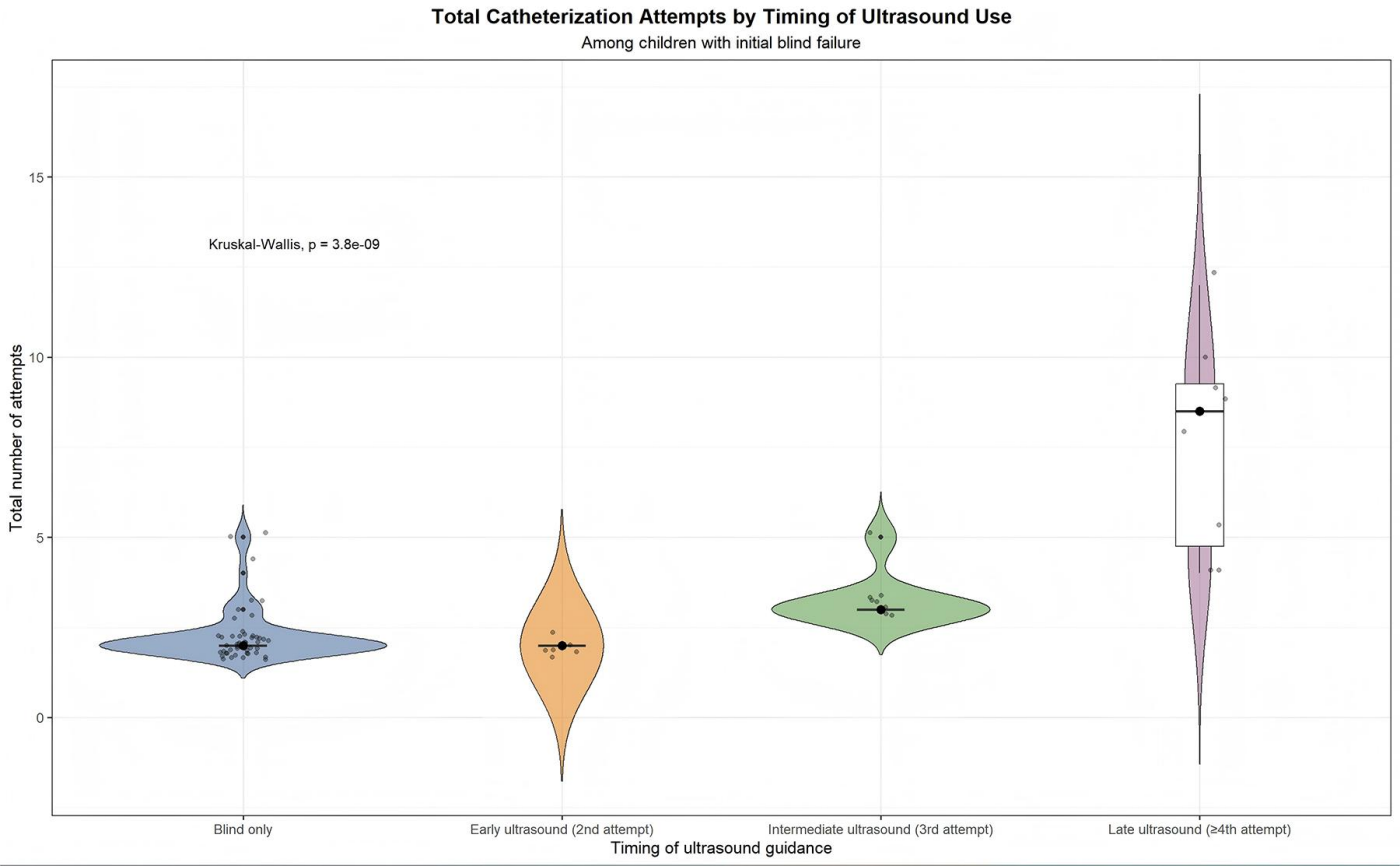
Attempts by timing of ultrasound guidance.

Ultrasound guiding timing affected the number of catheterization attempts in blind technique failure procedures (Figure 3). Descriptive statistics showed that earlier ultrasound guiding required fewer tries than delayed ultrasound rescue (Supplementary Table S2). Using early ultrasound rescue at the second try as the reference category, more efforts were needed for procedures with ultrasound guidance introduced after the fourth attempt (IRR = 3.81, 95% CI 2.05–7.08, *P* < .001). Blind-only operations and intermediate ultrasound use at the third try did not vary (IRR 1.14, 95% CI 0.63–2.07, *P* = .66 and 1.63, 95% CI 0.82–3.22, *P* = .16, Supplementary Table S3). With a dispersion ratio of 0.30, overdispersion was not evident.

## Discussion

This study examined 320 PAC procedures from prospectively collected clinical data from a PICU cohort with a 65% first-attempt success rate. First-attempt success was significantly correlated with puncture site, ultrasound-guided insertion, and neurological disease, as determined by multivariable analysis. Cannulation at the dorsalis pedis artery, ulnar artery, and other sites displayed lower success rates in comparison to the radial artery, whereas ultrasound guidance was substantially associated with a higher success rate. Additionally, fewer overall cannulation attempts were associated with an earlier conversion to ultrasound guidance after an initial unsuccessful landmark-guided attempt. Previous studies have found that first-attempt pediatric artery catheterization success rates range from 35% to 75% (12–14), depending on patient age, underlying diseases, operator expertise, and insertion strategies. Real-world observational studies in PICUs have similar success rates to this study.

The first-attempt success rates of PAC varied among different disease categories. Children with neurological disorders had a lower first-attempt success rate than those with respiratory conditions in the multivariable analysis. Nevertheless, it is important to exercise caution when interpreting this discovery. Neurological diseases include intracranial hemorrhage, status epilepticus, traumatic brain injury, and acute necrotizing encephalopathy vary in hemodynamic status, clinical urgency, and procedural context (15, 16). Additionally, the study did not evaluate vascular anatomical characteristics, including vessel diameter and profundity, which are recognized as predictors of successful cannulation (17). Consequently, the observed disparities between disease categories necessitate additional validation in future research.

Puncture site selection remained an independent determinant of successful PAC in critically ill children. Compared with the radial artery, catheterization at the dorsalis pedis, ulnar, and other peripheral arteries was associated with significantly lower success rates, indicating that site selection continues to play a decisive role even with ultrasound guidance (5, 18). The radial artery's superficial location, relatively consistent anatomy, and collateral circulation likely preserve arterial pulsatility and signal detectability under conditions common in the PICU, such as hypoperfusion and peripheral edema (19). Consistent with prior pediatric studies, these findings support the radial artery as the preferred first-line site, with alternative arteries considered only when radial access is not feasible (20).

Cannulation technique was found to be a key factor in PAC in critically ill children. Ultrasound guiding increased first-attempt success compared to landmark guidance, consistent with pediatric and critical care research (5, 18). The cumulative distribution of cannulation attempts indicated that the ultrasound-guided group had a consistently higher rate of successful catheterization within multiple attempts. Ultrasound guidance improves first-attempt success and reduces the number of attempts required for successful catheterization by providing real-time visualization of vascular anatomy, allowing clinicians to identify unfavorable conditions and adjust needle direction during puncture. Consistently, systematic reviews and meta-analyses have demonstrated higher first-attempt success and fewer cannulation attempts with ultrasound-guided arterial catheterization (12, 21). In critically ill children with hemodynamic instability, poor peripheral perfusion, or narrow vessels, ultrasound guidance may be particularly advantageous, as landmark-based techniques are frequently restricted.

Importantly, early conversion to ultrasound guidance after a failed landmark-guided attempt significantly reduced cannulation attempts, whereas delayed adoption after multiple failures increased procedural burden. This finding highlights the value of timely ultrasound rescue to minimize repeated punctures and procedure time.

No significant correlation was found between cannulation efficiency and PICS (22, 23), indicating that the overall severity of the illness may have a limited impact on the efficacy of arterial catheterization, which is consistent with previous reports (6). Conversely, the significance of vascular anatomical characteristics and local vessel conditions may be greater. Puncture site and specific disease categories were still associated with first-attempt success in the multivariable analysis, even after adjusting for cannulation technique.

From a clinical standpoint, these results are in favor of the preferential use of ultrasound guidance for PAC in the PICU, particularly in children with hemodynamic instability, weak peripheral pulses, or prior failed attempts. Early ultrasound guidance may enhance procedural efficiency.

## Limitations

Some limitations exist in this investigation. The findings of this single-center investigation in one PICU may not be generalizable and require multicenter validation. The majority of arterial catheterization was performed by qualified nurses with at least four years of PICU experience, but operator-level information (e.g., years of experience or previous procedural volume) was not recorded, preventing further evaluation of operator expertise. Second, residual confounding may occur in observational studies based on prospectively obtained data. Important vascular anatomical characteristics, including catheter-to-vessel ratio, vessel profundity, and vessel diameter, were not assessed. Third, procedure-related complications were not systematically recorded, and therefore the specific causes of catheterization failure, such as hematoma or minor bleeding, could not be further quantified. Lastly, in contrast to certain previous studies that defined first-attempt success as successful cannulation with a single needle insertion, this study allowed for limited needle redirections during a single skin penetration to more accurately reflect real-world PICU practice. Comparisons between studies may be impacted by this definition. Furthermore, the sample size for analyses of ultrasound rescue after failure landmark-guided attempts remained relatively small, and further investigation is warranted to determine the optimal rescue timing.

## Conclusion

The puncture site and insertion technique were identified as critical factors associated with the success of PAC on the first attempt in critically ill children in this real-world PICU study. The success rates were higher when radial artery access and ultrasound guidance were used, and the number of cannulation attempts may be reduced by early ultrasound rescue after failed landmark attempts. The procedural efficacy of PICU practice may be enhanced by optimizing site selection and ultrasound-guided strategies.

## Data Availability

The datasets supporting the conclusions of this article are included in the article and its Supplementary Material. Additional data are available from the corresponding authors upon reasonable request, subject to ethical and privacy restrictions involving pediatric patients.
