# Supplementary material for: Factors associated with first-attempt success of peripheral arterial catheterization in a pediatric intensive care unit: a prospective observational study
Source: Front Pediatr. 2026 Apr 23;14:1802930. doi: 10.3389/fped.2026.1802930 (PMC13149438; doi:10.3389/fped.2026.1802930)
Supplement: Supplementary file 1 [file Table1.docx]

Supplementary：Table S1 Multicollinearity diagnostics for variables included in the multivariable model

| Variable | GVIF | Df | GVIF^(1/(2*Df)) |
| --- | --- | --- | --- |
| insertion_site | 1.42 | 3 | 1.06 |
| diagnosis | 2.28 | 5 | 1.09 |
| age_group | 2.58 | 4 | 1.13 |
| gender | 1.10 | 1 | 1.05 |
| PICS_10 | 1.23 | 1 | 1.11 |
| MAP_10 | 1.29 | 1 | 1.14 |
| catheter_size | 1.57 | 1 | 1.25 |
| first_technique | 1.17 | 1 | 1.08 |
| mechanical_ventilation | 1.44 | 1 | 1.20 |
| compliance | 1.22 | 1 | 1.10 |
| PLT_50 | 1.24 | 1 | 1.12 |
| INR_1 | 1.23 | 1 | 1.11 |

Supplementary：Table S2. Descriptive statistics of total catheterization attempts by timing of ultrasound use

| ultrasound_timing | n | Mean_SD | Median_IQR |
| --- | --- | --- | --- |
| Early ultrasound (2nd attempt) | 6 | 2.00 ± 0.00 | 2.00 (2.00, 2.00) |
| Blind only | 46 | 2.28 ± 0.72 | 2.00 (2.00, 2.00) |
| Intermediate ultrasound (3rd attempt) | 8 | 3.25 ± 0.71 | 3.00 (3.00, 3.00) |
| Late ultrasound (≥4th attempt) | 8 | 7.62 ± 2.97 | 8.50 (4.75, 9.25) |
| Note：Reference group: Early ultrasound (2nd attempt) | | | |

Supplementary：Table S3. Poisson regression for total catheterization attempts according to ultrasound timing

| Variable | IRR (95%CI) | *P* value |
| --- | --- | --- |
| Blind only vs Early ultrasound (2nd attempt) | 1.14 (0.63, 2.07) | .664 |
| Intermediate ultrasound (3rd attempt) vs Early ultrasound (2nd attempt) | 1.63 (0.82, 3.22) | .164 |
| Late ultrasound (≥4th attempt) vs Early ultrasound (2nd attempt) | 3.81 (2.05, 7.08) | <.001 |
